# Association of ventilator type with hospital mortality in critically ill patients with SARS-CoV2 infection: a prospective study

**DOI:** 10.1186/s13613-022-00981-2

**Published:** 2022-02-08

**Authors:** Alexis Ferré, Fabien Marquion, Marc Delord, Antoine Gros, Guillaume Lacave, Virginie Laurent, Sybille Merceron, Marine Paul, Christelle Simon, Gilles Troché, Clément Charbonnel, Stéphanie Marque-Juillet, Fabrice Bruneel, Stéphane Legriel, the RESPI-COVID19 Study Group, Sofia Abbad, Georges Abi Abdallah, Passem Ahmed, Marlène Amara, Marine Arrayago, Alix Aubry, Pauline Bargain, Jean-Pierre Bédos, Hugo Bellut, Michael Benayoun, Hotman Benhamida, Laura Benchetrit, Johan Benhard, Emilie Boglietto, Raphaelle Bordier, Antoine Brizard, Amélie Cambriel, Steven Causeret, Raphaële Convers-Domart, Paul Chinardet, Anaïs Codorniu, Adrien Coeffic, Wandrille de Carrere, Cyril Dekeyser, Alix Delaroche, Chloé Descamps, Juliette Didier, Pascaline Dorges, Lucie Fanet, Camille Fauquenot, Claire Flaujac, Laura Gouzien, Louis Grandière, Juliana Henao-Brasseur, Jean-Didier Heymann, Charles Hickel, Philippe Jullien, Myriam Lamamri, Bénédicte Le Clec’h, Marc Lessert, Yves Le Tulzo, Bernard Livarek, Aurélien Maurizot, Céline Metzger, Hervé Michon, Marie-Sophie Minin, Ghislane Nid-Bella, Marianne Offredo, Amael Ouassou, Hanna Paktoris, François Perier, Olivia Picq, Hélène Poirier, Jean-Herlé Raphalen, Anne Roche, Ariane Roujansky, Thomas Quenesson, Jil Rouaux, Lucie Sabau, Marie Saleten, Marie Salvetti, Florence Sarfati, Pierre Squara, Celia Teissedre, Manon Terris, François Stephan, Fabienne Tamion, Jean-François Vax, Benoît Veber, Cécile Vernet, Alexandre Wormser

**Affiliations:** 1 Intensive Care Unit, Versailles Hospital, 177 Rue de Versailles, 78150 Le Chesnay, France; 2 Department of Anesthesiology, Versailles Hospital, Le Chesnay, France; 3 Clinical Research Center, Versailles Hospital, Le Chesnay, France; 4 Present Address: Department of Population Health Sciences, Faculty of Life Sciences & Medicine, King’s College London, London, UK; 5 Department of Cardiology, Versailles Hospital, Le Chesnay, France; 6 Department of Virology, Versailles Hospital, Le Chesnay, France; 7 University Paris-Saclay, UVSQ, INSERM, CESP, “PsyDev” Team, Villejuif, France

**Keywords:** COVID-19, ICU, Ventilator, Mortality, Outcomes

## Abstract

**Background:**

To evaluate the association between ventilator type and hospital mortality in patients with acute respiratory distress syndrome (ARDS) related to COVID-19 (SARS-CoV2 infection), a single-center prospective observational study in France.

**Results:**

We prospectively included consecutive adults admitted to the intensive care unit (ICU) of a university-affiliated tertiary hospital for ARDS related to proven COVID-19, between March 2020 and July 2021. All patients were intubated. We compared two patient groups defined by whether an ICU ventilator or a less sophisticated ventilator such as a sophisticated turbine-based transport ventilator was used. Kaplan–Meier survival curves were plotted. Cox multivariate regression was performed to identify associations between patient characteristics and hospital mortality. We included 189 patients (140 [74.1%] men) with a median age of 65 years [IQR, 55–73], of whom 61 (32.3%) died before hospital discharge. By multivariate analysis, factors associated with in-hospital mortality were age ≥ 70 years (HR, 2.11; 95% CI, 1.24–3.59; *P* = 0.006), immunodeficiency (HR, 2.43; 95% CI, 1.16–5.09; *P* = 0.02) and serum creatinine ≥ 100 µmol/L (HR, 3.01; 95% CI, 1.77–5.10; *P* < 0.001) but not ventilator type. As compared to conventional ICU (equipped with ICU and anesthesiology ventilators), management in transient ICU (equipped with non-ICU turbine-based ventilators) was associated neither with a longer duration of invasive mechanical ventilation (18 [IQR, 11–32] vs. 21 [13–37] days, respectively; *P* = 0.39) nor with a longer ICU stay (24 [IQR, 14–40] vs. 27 [15–44] days, respectively; *P* = 0.44).

**Conclusions:**

In ventilated patients with ARDS due to COVID-19, management in transient ICU equipped with non-ICU sophisticated turbine-based ventilators was not associated with worse outcomes compared to standard ICU, equipped with ICU ventilators. Although our study design is not powered to demonstrate any difference in outcome, our results after adjustment do not suggest any signal of harm when using these transport type ventilators as an alternative to ICU ventilators during COVID-19 surge.

**Supplementary Information:**

The online version contains supplementary material available at 10.1186/s13613-022-00981-2.

## Take home message

During COVID-19 pandemics when many healthcare systems were overwhelmed and ICUs saw huge surges in admissions, using sophisticated turbine-based transport ventilators after admission was associated neither with higher in-hospital mortality nor with longer invasive mechanical ventilation duration in critically ill patients with SARS-CoV2 infection-related ARDS. Although our study design is underpowered to demonstrate outcome differences, our results do not suggest any signal of harm when using these transport type ventilators as an alternative to ICU ventilators during COVID-19 surge.

## Background

The current COVID-19 pandemic, caused by the SARS-CoV2 virus, is evolving in waves that put healthcare systems under severe strain [1–4]. Nearly 10% of patients with COVID-19 have severe acute respiratory distress syndrome (ARDS) that requires admission to the intensive care unit (ICU) and, in many cases, invasive mechanical ventilation (MV) [5–9]. As a consequence, waves of COVID-19 often stretch the available ICU resources far beyond their intended limits [10]. Transient ICU beds must then be created in operating rooms, emergency departments, and other parts of the hospital. These beds need to be equipped, most importantly with competent staff and with ventilators.

The management of ARDS relies on lung-protective ventilation according to international recommendations [11]. Not all existing ventilators have the technical capabilities required for optimal lung-protective ARDS ventilation, nor the possibility of close monitoring the plateau and driving pressures and compliance. In many hospitals, surges in ICU admissions during waves of COVID-19 result in a shortage of sophisticated ICU ventilators. To fill this gap, simpler ventilators such as those designed for patient transport are used. These simpler transport ventilators could be less efficient for the treatment of ARDS, because their intrinsic performances and/or lung monitoring may be insufficient. Moreover, physiological studies have demonstrated an influence of ventilator type on patient comfort, work of breathing, and patient-ventilator asynchronies, with considerable variations across ventilator models [12]. An investigation of the potential association between the use of transport ventilators and the survival of patients with COVID-19-related ARDS was, therefore, timely.

The primary objective of this study was to look for an association linking the type of ventilator used (ICU ventilators, including conventional ICU- and anesthesia ventilator vs. sophisticated turbine-based transport ventilators) and hospital mortality in patients requiring MV for COVID-19-related ARDS. The secondary objectives were to look for associations linking ventilator type to invasive MV duration, ventilation outcomes (prone positioning, rescue inhaled nitric oxide and extracorporeal membrane oxygenation [ECMO]), ICU-length of stay and day-90 mortality.

## Methods

This single-centre observational prospective study was approved by the ethics committee of the French Intensive Care Society (#20-42) and registered at the French National Institute for Health Data (#MR 4109060520). Informed consent was sought from the next of kin, if available, and from the patients upon recovery of competency, in compliance with French law.

### Patients

Consecutive adults admitted between March 5, 2020 and July 6, 2021, to one of the four Versailles hospital ICUs for severe proven SARS-CoV2 infection were prospectively included in the RESPI-COVID19 registry. For the analysis of this study, only patients with ARDS related to severe SARS-CoV2 infection requiring invasive MV were eligible. Patients who received MV for causes other than respiratory failure (e.g., non-hypoxic cardiac arrest, cardiogenic or septic shock, neurological disorder, or pregnancy-related disease) were not included.

### Study setting and COVID-19 surge

The Versailles Hospital is a university-affiliated tertiary hospital in the Paris area with 800 medical and surgical beds. The 28-bed closed ICU has 20 ICU beds and 8 intermediate-care beds for continuous monitoring. In March 2020, the first wave of COVID-19 produced a sudden and massive increase in the numbers of patients with ARDS requiring critical care. Transient ICU beds for COVID-19 patients were set up in the intermediate-bed unit, the post-anesthesia care units, and the cardiology ICU. The total number of ICU beds was then 49, in four different places in the hospital. ICU ventilators were available for 27 beds. The remaining 22 beds were equipped with non-ICU (transport) ventilators. During the second and the third wave, transient ICU beds for COVID-19 patients were set up once again.

Dedicated ICU teams (paramedics and physicians) operated in usual ICU unit and the transient ICU from the intermediate-bed unit (28 beds). Anesthesiologist teams (paramedics and physicians) operated in the transient ICUs from the post-anesthesia care units (15 beds). A written protocol for COVID-19 related ARDS management was disseminated at the start of the epidemic and then regularly updated (according to scientific knowledge and guidelines at this time). The ICU team participated to dedicated staff in the transient ICUs.

The choice of hospitalization ICU area was first made based on bed availability and on individual patient renal support needs as renal replacement therapy was available only in the conventional ICU.

### Definitions

Proven SARS-CoV2 infection was defined as a positive reverse transcriptas-polymerase chain reaction (RT-PCR) result for SARS-CoV2 obtained on a respiratory tract sample (nasal or nasopharyngeal swab, tracheal aspirate, or bronchial aspirate).

We defined two groups of patients based on ventilator type according to published comparative bench test results [13–18]. The ICU group, so called ICU-ventilator, comprised the high-performance devices used in our ICUs (e.g., Evita XL or Evita Infinity C500^®^, Dräger, Lübeck, Germany; Carescape R860^®^, General Electrics Healthcare, Boston, MA, USA) or in operating rooms (e.g., Aisys CS2^®^, Datex Ohmeda, General Electrics Healthcare). The non-ICU group, so-called transport-ventilator, comprised sophisticated turbine-based ventilators generally designed for mobile interventions but used in transient ICUs set up to handle the surge of patients with severe COVID-19 (e.g., Monnal T60 and T75^®^, Air Liquide Healthcare, Paris, France; Elisée 350 and 250^®^, ResMed, Saint-Priest, France). Because of the surge, bed availability, opening and then closing transient ICUs to face the evolving crisis, some patients were ventilated with both ventilator type during the study period. For these patients, classification group was defined according to composite criteria regarding ventilator type used during the first 10 days of the acute phase of ARDS management, and the ratio period time of the entire invasive MV duration for the eligible ventilator type. Heat and moisture exchanger were used during wave 1 then heated humidifiers during wave 2 and 3.

### Standardized ICU management of patients with COVID-19-related ARDS

The same standardized care management was used at all four ICU sites. At the early phase of the pandemic (first wave), whether virus aerosolization could occur with non-invasive ventilation (NIV) and/or high-flow nasal oxygen (HFNO) was unknown. Consequently, conventional oxygen therapy via a face mask was the preferred, albeit not exclusive, modality of initial oxygen administration [19]. The criteria for invasive MV initiation were persistent severe hypoxemia (SpO_2_ < 90% despite 12–15 L/min face-mask oxygen or FiO_2_ = 100% on NIV or HFNO) or persistent clinical signs of acute respiratory distress. Patients with ARDS as defined by the Berlin classification received invasive MV (volume assist controlled ventilation mode, tidal volume of 6 mL/kg of predicted body weight, positive end-expiratory pressure regarding FiO_2_ level with respect to plateau pressure ≤ 28–30 cmH_2_O), sedative drugs, neuromuscular blockade and prone positioning in compliance with international guidelines [11, 20–23]. Inhaled nitric oxide and/or recruitment maneuvers were performed as rescue therapy if deemed necessary by the physician in charge. The appropriateness of using rescue ECMO was discussed collegially with referral center specialists (ICU, Pitié-Salpêtrière Teaching Hospital, Paris, France) in all patients with prespecified criteria [24–26]. Prophylactic anticoagulation was given in a standard dosage based on patient weight until March 25 and in an intermediate dosage thereafter [27–29]. Bacterial co-infection at admission or during the ICU stay was carefully screened for and treated as appropriate.

Since summer 2020 and the positive results of the RECOVERY study on steroids efficacy for hypoxemic SARS-CoV2 patients [30], international guidelines had recommended [31] their use for all critical patients requiring supplemental oxygen. Patients admitted since July 25, 2020 received intravenous dexamethasone 6 mg per day for 10 days. In addition, preliminary data on risk contamination (viral aerosolization) leads to reassurance for non-invasive ventilatory assistance, such as HFNO and face-mask NIV [32–34]. At this time, we used HFNO as first line oxygen therapy for all patients admitted since July 25, 2020 for a de novo acute respiratory failure related to a proven SARS-CoV2 infection.

Rescue intravenous dexamethasone therapy, 20 mg/day for 5 days followed by 10 mg/day for 5 days could be initiated for patients with persistent moderate-to-severe ARDS (PaO_2_/FiO_2_ < 150) and low respiratory static compliance (< 30 mL/cmH_2_O) despite ventilator setting optimization, intravenous neuromuscular blocking agent infusion, and prone positioning [20, 35, 36].

### Data collection

Data for each patient were collected into an electronic file (Excel®, Microsoft, Redmond, WA) whose access was restricted by a code known only by the data collector [AF]. The data files were anonymized by assigning a number to each patient. We collected demographic characteristics and comorbidities. Clinical and laboratory findings at ICU admission were recorded. The last PaO_2_/FiO_2_ ratio before intubation was calculated using the following formula for patients without NIV or HFNO: PaO_2_/(FiO_2_ = 0.21 + 0.03 × O_2_ in L/min delivered nasally or through a face-mask) [37]. The following data were also collected: severity and description of organ failures according to the Simplified Acute Physiology score II (SAPS-II) [38] and the Sepsis-related Organ Failure Assessment (SOFA) score [39], the use of prone positioning, inhaled nitric oxide, vasoactive drugs, renal replacement therapy, and ECMO.

We recorded the ventilator device used from ICU admission to ICU discharge, total MV duration, and total NIV and/or HFNO duration if relevant. Finally, we collected ICU and hospital lengths of stay, ICU mortality, in-hospital mortality, and day-90 mortality (based on hospital medical records and follow-up, rehabilitation-care unit data, or general practitioner data at day-90).

### Statistical analysis

Quantitative parameters were described as median [interquartile range] and qualitative parameters as number (percentage). We compared categorical variables using Fisher’s exact tests and continuous variables using Wilcoxon rank-sum tests.

Survival curves were obtained using the Kaplan–Meier estimator and data were censored at day-90 according to vital status. To identify associations between patient characteristics and hospital mortality, a Cox proportional hazard analysis was performed for each variable. A multivariate model was then built with variables that yielded *P* values smaller than 0.05 by univariate analysis and/or were clinically relevant according to the present study or previous published data on severe COVID-19 (ventilator type, age, immunodeficiency, last PaO_2_/FiO_2_ ratio before invasive mechanical ventilation, creatininemia at ICU admission, dexamethasone initiated at ICU admission). Missing data were imputed using multivariate imputation by chained equations.

To better evaluate the effect of ventilator exposure on hospital mortality, we conducted an ancillary propensity score matching analysis. Used variables (age, male sex, time from hospitalization to ICU admission, SAPS II, respiratory SOFA score and hepatic SOFA score, creatinine at ICU admission ≥ 100 µmol/L and LDH value at ICU admission) were associated to both ventilator exposure and outcome [40]. Matching tended to reach 1:2 given a 0.28 caliper on the logit of the propensity score [41].

All tests were two-sided and *P* values < 0.05 were considered significant. Analyses were performed using the R statistical program version 4.0.3 (R Foundation for Statistical Computing, Vienna, Austria).^1^

## Results

### Patients

**Fig. 1:**
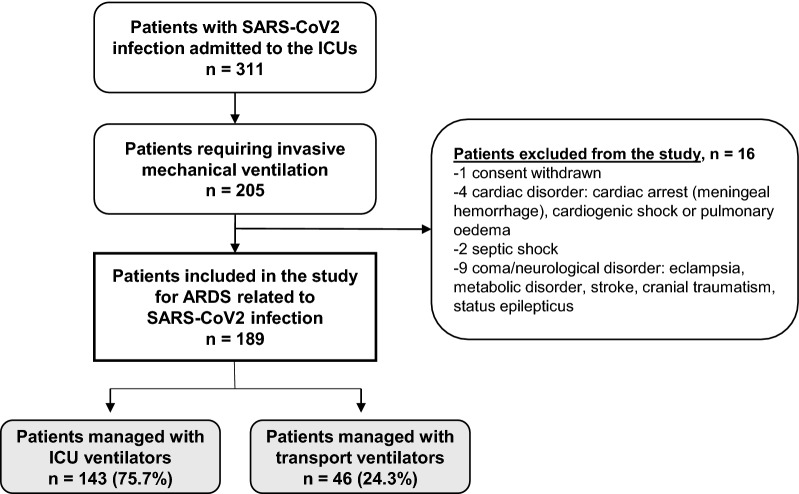
Patient flowchart. *SARS-CoV2* severe acute respiratory syndrome-coronavirus type 2, *ARDS* acute respiratory distress syndrome, *ICU* intensive care unit

Figure 1 shows the patient flowchart. Of the 311 critically ill patients with a positive RT-PCR for SARS-CoV2 managed in the four COVID-19 ICUs between March 5, 2020 and July 6, 2021, 189 were included in the study.

Table 1 reports the main patients characteristics. There were 140 (74.1%) men with a median age of 65 [IQR, 57–73] years. Obesity (BMI ≥ 30 kg/m^2^) was present in 70 (37.2%) patients and a history of arterial hypertension and diabetes mellitus in 87 (46%) and 55 (29.1%) patients, respectively. Median time from symptom onset to ICU admission was 9 [IQR, 6–11] days.

**Table 1:** Baseline and ICU admission characteristics according to ventilator type in patients with acute respiratory distress syndrome due to COVID-19

Variables	All patients *n* = 189	ICU ventilator *n* = 143 (75.7)	Transport ventilator *n* = 46 (24.3)	*P* value		*N* missing^a^
Demographic characteristics						
Age (years)	65 [57–73]	65 [56–74]	65 [60–73]	0.90		
Male sex	140 (74.1)	112 (78.3)	28 (60.9)	0.03		
Active smoking	11 (6.0)	9 (6.4)	2 (4.4)	1.00		4
Comorbidities						
Coronary artery disease	18 (9.5)	14 (9.8)	4 (8.7)	1.00		
Treated arterial hypertension	87 (46.0)	69 (48.3)	18 (39.1)	0.31		
Diabetes mellitus	55 (29.1)	45 (31.5)	10 (21.7)	0.26		
Immunodeficiency	18 (9.5)	15 (10.5)	3 (6.5)	0.57		
Obesity (BMI ≥ 30 kg/m^2^)	70 (37.2)	56 (39.4)	14 (30.4)	0.30		1
Asthma	15 (7.9)	10 (7.0)	5 (10.9)	0.37		
COPD	10 (5.3)	8 (5.6)	2 (4.4)	1.00		
Bronchectasis	2 (1.1)	2 (1.4)	0 (0.0)	1.00		
Characteristics at ICU admission						
Epidemic COVID-19 wave				0.23		
Wave 1	82 (43.4)	66 (46.2)	16 (34.8)			
Wave 2	37 (19.6)	29 (20.3)	8 (17.4)			
Wave 3	70 (37.0)	48 (33.6)	22 (47.8)			
SAPS II	40 [34–49]	41 [34–50]	40 [35–45]	0.45		
Day 1, total SOFA score^b^	6 [4–7]	6 [4–7]	6 [4–7]	0.72		1
Respiratory SOFA score	3 [3, 4]	3 [3, 4]	3 [-4]	0.18		
Cardiovascular SOFA score	3 [0–3]	3 [0–3]	3 [0–3]	0.96		
Renal SOFA score	0 [0–0]	0 [0–1]	0 [0–0]	0.28		1
Neurological SOFA score	0 [0–0]	0 [0–0]	0 [0–0]	0.89		
Hepatic SOFA score	0 [0–0]	0 [0–0]	0 [0–0]	0.13		1
Coagulation SOFA score	0 [0–0]	0 [0–0]	0 [0–1]	0.28		1
Time from symptom onset to ICU admission (days)	9 [6–11]	8 [6–12]	9 [6–11]	0.84		1
Time from hospitalization to ICU admission (days)	1 [0–3]	1 [0–3]	2 [0–4]	0.04		1
Pulmonary co-infection at ICU admission	16 (8.5)	13 (9.1)	3 (6.5)	0.70		
Oxygen requirements at ICU admission (L/min)	15 [15–15]	15 [15–15]	15 [13–15]	0.71		4
Laboratory tests at ICU admission						
Lactate (mmol/L)	1.5 [1.2–1.9]	1.6 [1.3–2.0]	1.4 [1.2–1.7]	0.03		1
LDH (IU/L)	724 [532–915]	759 [609–1002]	580 [464–763]	< 0.001		3
Lymphocytes (G/L)	0.66 [0.45–0.95]	0.70 [0.45–1.00]	0.62 [0.43–0.84]	0.17		6
C-reactive protein (mg/L)	167 [94–242]	170 [95–245]	147 [86–200]	0.25		23
Procalcitonin (ng/mL)	0.4 [0.2–1.1]	0.4 [0.2–1.3]	0.3 [0.2–0.7]	0.16		26
d-dimers (ng/mL)	1525 [883–2550]	1750 [970–2650]	1090 [770–1730]	0.02		27
Creatinine (µmol/L)	73 [60–98]	78 [62–101]	66 [53–88]	0.02		1
Troponin (ng/mL)	0.01 [0.01–0.02]	0.01 [0.01–0.03]	0.01 [0.01–0.02]	0.44		25
NT-proBNP (pg/mL)	383 [156–973]	473 [188–1021]	311 [143–670]	0.27		70

Data are Presented as *N* (%) or median [interquartile range]

*BMI* body mass index, *COPD* chronic obstructive pulmonary disease, *ICU* intensive care unit, *SAPS II* Simplified Acute Physiology Score II, *SOFA* sepsis-related organ failure assessment, *LDH* lactate dehydrogenase, *CRP* NT-proBNP: NT-pro B-type natriuretic peptide

^a^Number of missing observations, unless Ø

^b^Six organs or systems are assessed, each receiving 0 (no dysfunction) to 4 points (more severe dysfunction). The sum of scores ranges from 0 to 24; higher scores indicate more severe disease

### ICU management

At ICU admission, 16 (8.5%) of the 189 patients received orotracheal intubation and MV before ICU admission because of on-scene life-threatening respiratory distress. The remaining 173 non- intubated patients before ICU admission received face-mask supplemental oxygen at a median rate of 15 [IQR, 15–15] L/min; of these, 112 (59.3%) were switched to HFNO after ICU admission and 48 (25.4%) to supplemental NIV. All patients received MV with a median time from ICU admission to intubation of 0 [IQR, 0–1] days. The last PaO_2_/FiO_2_ ratio before intubation was 89 [IQR, 71–123], corresponding to a median FiO_2_ of 80% [IQR, 66%-100%].

### Comparison according to ventilator type

Of the 189 patients, 143 (75.7%) were managed with ICU ventilators and 46 (24.3%) with transport ventilators (Table 1). The only baseline differences between the two groups were for male sex percentage, time from hospitalization to ICU admission, lactate, LDH, D-dimers and serum creatinine. The two groups were not significantly different in terms of severity scores during the first 24 h after ICU admission (SAPS II and specific organ SOFA scores) neither for the use of prone positioning, number of prone position sessions per patient, rescue use of inhaled nitric oxide, rescue use of ECMO, or frequency of ventilator-associated pneumonia (Table 2).

**Table 2:** ICU management and outcomes according to ventilator type in patients with COVID-19-related acute respiratory distress syndrome

Variables	All patients *n* = 189	ICU ventilator *n* = 143 (75.7)	Transport ventilator *n* = 46 (24.3)	*P* value	*N* missing^a^
ICU management					
High-flow nasal oxygen	112 (59.3)	80 (55.9)	32 (69.6)	0.12	
Non-invasive ventilation	48 (25.4)	33 (23.1)	15 (32.6)	0.24	
Last PaO_2_/FiO_2_ ratio before intubation	89 [71–123]	89 [72–123]	84 [69–113]	0.52	28
Ventilatory settings, first day of ARDS					
First PaO_2_/FiO_2_ ratio under invasive MV	139 [99–178]	139 [99–178]	126 [98–174]	0.36	2
Corresponding first FiO_2_ under invasive MV	1.0 [0.8–1.0]	1.0 [0.8–1.0]	0.9 [0.7–1.0]	0.02	2
Tidal volume (mL/kg PBW)	6.0 [5.9–6.2]	6.0 [5.9–6.2]	6.0 [6.0–6.1]	0.64	3
Set PEEP, cm H_2_O	12 [10–12]	12 [10–12]	12 [10–12]	0.69	2
Plateau pressure (P_PLAT_, cm H_2_O)	23 [21–25]	23 [21–25]	22 [20–24]	0.23	25
Driving pressure, cm H_2_O	10 [9–12]	11 [9–13]	10 [9–11]	0.10	25
Time from ICU admission to intubation (days)	0 [0–1]	0 [0–1]	0 [0–1]	0.34	1
Prone positioning	133 (70.4)	99 (69.2)	34 (73.9)	0.58	
Number of prone position sessions	4 [2–7]	4 [2–8]	4 [2–6]	0.43	
Inhaled nitric oxide	52 (27.5)	42 (29.4)	10 (21.7)	0.35	
ECMO	10 (5.3)	10 (7.0)	0 (0.0)	0.12	
Ventilator-associated pneumonia	110 (58.2)	80 (55.9)	30 (65.2)	0.31	
Weaning failure (≥ 2 orotracheal intubations)	27 (14.3)	17 (11.9)	10 (21.7)	0.14	
Tracheostomy	27 (14.3)	20 (14.0)	7 (15.2)	0.81	
Need for vasoactive drugs in the ICU	176 (93.1)	134 (93.7)	42 (91.3)	0.52	
Need for renal replacement therapy in the ICU	28 (14.8)	25 (17.5)	3 (6.5)	0.09	
Dexamethasone initiated at ICU admission	107 (56.6)	77 (53.9)	30 (65.2)	0.23	
Rescue dexamethasone for persistent ARDS	38 (20.1)	30 (21.0)	8 (17.4)	0.68	
Outcomes					
Duration of invasive MV, days	19 [11–33]	18 [11–32]	21 [13–37]	0.39	
ICU length of stay (days)	24 [15–41]	24 [14–40]	27 [15–44]	0.44	
ICU mortality	60 (31.8)	47 (32.9)	13 (28.3)	0.59	
Post-ICU hospital length of stay (days)	10 [7–15]	11 [8–16]	9 [7–12]	0.10	
Hospital mortality	61 (32.3)	48 (33.6)	13 (28.3)	0.59	
Rehabilitation-unit length of stay (days)	27 [17–41]	27 [17–41]	31 [21–42]	0.68	
Day-90 mortality	63 (33.3)	50 (35.0)	13 (28.3)	0.47	

Data are presented as *N* (%) or Median [interquartile range]

*ICU* intensive care unit, *PaO*_*2*_*/FiO*_*2*_ partial pressure of oxygen to fraction of inspired oxygen, *ARDS* acute respiratory distress syndrome, *PBW* predicted body weight, *MV* mechanical ventilation, *PEEP* positive end-expiratory pressure, *ECMO* extracorporeal membrane oxygenation, *ARDS* acute respiratory distress syndrome

^a^Number of missing observations, unless Ø

### Determinants of hospital mortality

**Fig. 2:**
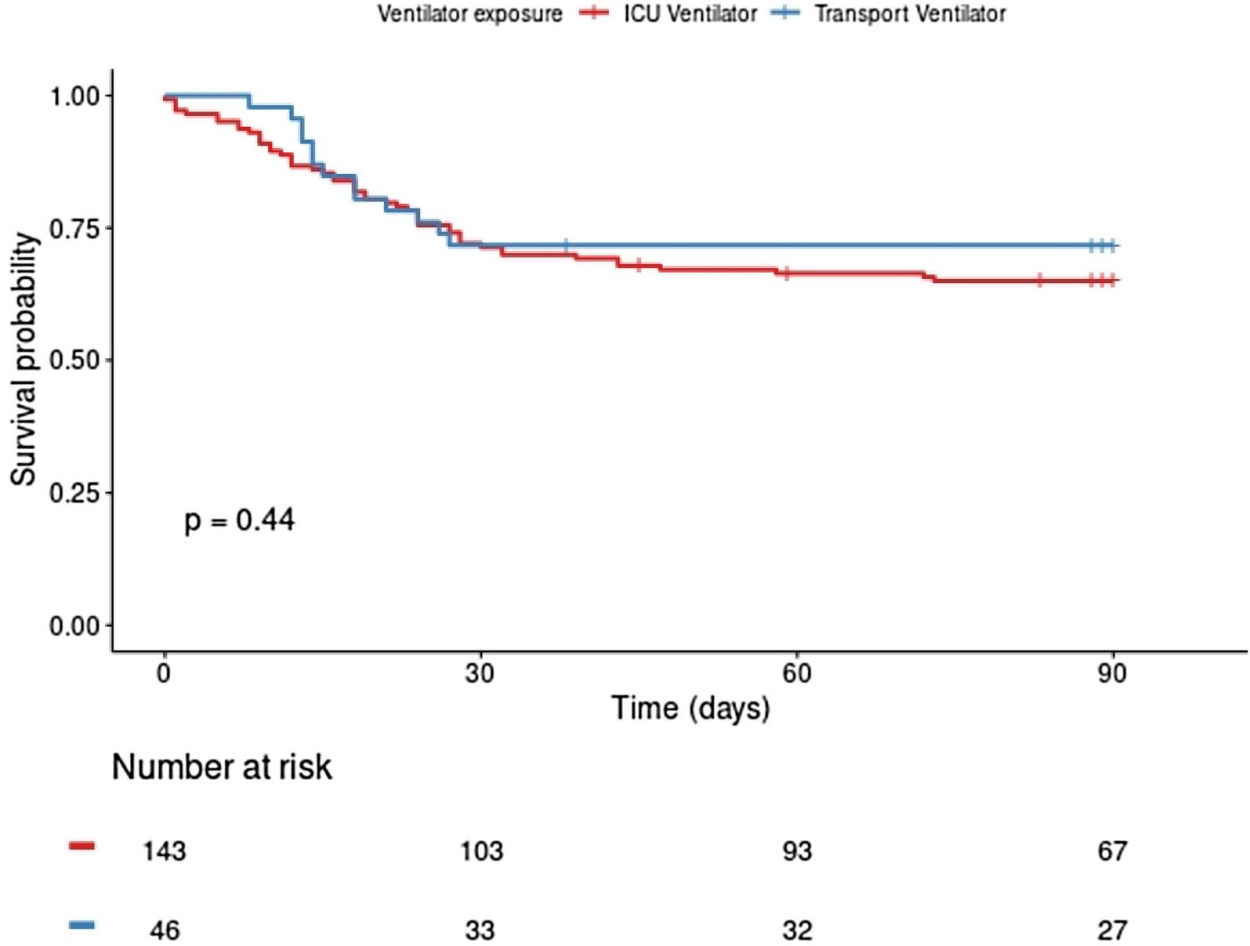
Kaplan–Meier survival curves according to ventilator type in 189 patients with COVID-19-related acute respiratory distress syndrome. *ICU* intensive care unit

**Fig. 3:**
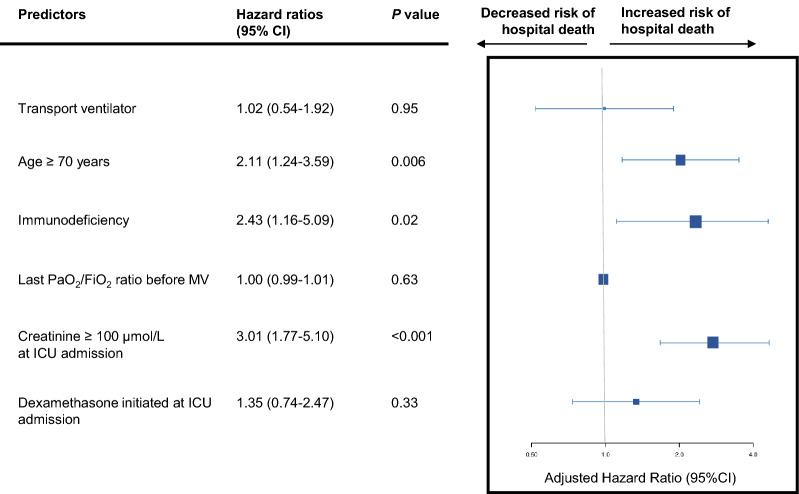
Multivariate analysis and forest plot: association of ventilator type and risk factors with risk of hospital mortality. Data marker sizes reflect the relative size of each covariate. Hazard ratios were computed after adjustment on the SAPS II. Error bars indicate 95% confidence intervals of hazard ratios. 95% CI denotes 95% confidence interval. *ICU* intensive care unit

Figure 2 reports the Kaplan–Meier survival curves in the two ventilator-type groups. Overall in-hospital mortality was 32.3% (61/189) with no significant between-group difference (*P* = 0.44). Results of univariate analysis of the variables associated with hospital mortality are shown in Additional file 1: Table S1. Ventilator type was not associated with hospital mortality in univariate analysis. By multivariate analysis (Fig. 3), the variables significantly associated with hospital mortality were age ≥ 70 years (HR, 2.11; 95%CI, 1.24–3.59; *P* = 0.006), immunodeficiency (HR, 2.43; 95%CI, 1.16–5.09; *P* = 0.02) and serum creatinine ≥ 100 µmol/L (HR, 3.01; 95%CI, 1.77–5.10; *P* < 0.001) but not ventilator type (ICU vs. transport).

The results of hospital mortality comparison between waves according to ventilator type did not show significant difference: 30% vs. 19% during wave 1 (*P* = 0.5), 34% vs. 38% during wave 2 (*P* > 0.9) and 38% vs. 32% during wave 3 (*P* = 0.6), respectively, for ICU-ventilator group and transport-ventilator group.

The results subset of the propensity score matching analysis was composed of 111 patients, whom 70 were exposed to ICU ventilators and 41 to transport ventilators. This sensitivity analysis produced comparable results without significant difference in hospital mortality rate (30% in the ICU ventilator group vs. 26.8% in the transport ventilator group, *P* = 0.83) (Additional file 1: Table S2).

### Other outcomes

Table 2 reports the patient outcomes in the two ventilator-type groups. MV duration, ICU mortality, hospital mortality, and day-90 mortality showed no significant between-group differences.

All patients were followed up until day-90.

## Discussion

To our knowledge, this study is the first to provide detailed information regarding ICU outcomes according to the ventilator type used in patients with COVID-19-related ARDS managed during epidemic spikes. Using less sophisticated ventilators instead of ICU ventilators in transient ICU was not significantly associated with ICU, in-hospital, or day-90 mortality. Neither was there any between-group difference in MV duration or in-ICU or hospital lengths of stay. However, our study focused only on two turbine-based non-ICU ventilators.

At the time of our study (March 2020 to July 2021), no etiological treatment had demonstrated efficacy against severe COVID-19. Consequently, the treatment relied on ventilatory assistance, other organ-supportive interventions, and symptomatic anti-inflammatory medications [30]. We focused on patients admitted for COVID-19-related ARDS requiring invasive MV. Of 311 patients admitted to our four ICUs, 66% were intubated in keeping with previous reports of proportions ranging from 76 to 87% during the first wave [42–47] and 56% for patients with high flow nasal oxygen failure [48].

The hospital mortality rate in our study of patients requiring MV was 32.3%, i.e., lower than in previous studies of populations with similar baseline characteristics [49, 50]. Differences in mortality may be related to differences in selection criteria for ICU admission and/or in bed availability [46]. Median MV duration was 19 days in our study, as compared to 12 days in previous French ICU study [45]. This longer MV duration may have resulted in extubation after the end of virus shedding and/or of clinically significant lung inflammation. Severity scores were generally similar across cohorts, with a median SAPSII of 40 in our study (including only patients requiring invasive MV) and 37 in a French nationwide cohort with 38% hospital mortality [45].

Our study suggests that, when no ICU ventilators are available, the use of sophisticated turbine-based transport ventilators to treat patients with COVID-19-related ARDS may be a valid alternative in transient ICU. Lung protective ventilation with low tidal volume and close lung monitoring is crucial to minimize alveolar damage and mortality in ARDS patients. Artificial ventilators must be able to provide lung protective ventilation during ARDS. Among the ventilators available on the market, especially transport ICU ventilators, not all have the technical performances to achieve such a result. In our study, we classified as ICU-ventilators the anesthesia ventilator Aisys CS2^®^ (Datex Ohmeda). Even if intrinsic performances are stated as close to standard ICU-ventilators, previous study testing the delivery of tidal volume from anesthesia ventilators during volume-controlled ventilation showed different volume error depending on set tidal volume, compliance, resistance, and delivered fresh gas flow [15]. For the Aisys CS2^®^, results showed a mean of 8–9% volume error (compliance set to 30 mL cm^−1^H_2_O) which could lead to unprotective ventilation. Lyazidi et al*.* showed a difference between preset and delivered tidal volume (1–2 mL/kg) despite intrinsic compensation algorithms in ICU ventilators [16]. The absolute difference was lower but not zero (< 5%) for Evita XL^®^ (Dräger) used in our study. Regardless of the type of ventilators used, we can observe a difference between performances and delivered ventilation despite optimal settings. However, no test can determine the clinical relevant effect of these error margins. In our study, we evaluated only two sophisticated turbine-based ventilators as transport-ventilators. L’Her et al*.* classified emergency and transport ventilators according to their performances and technical reliability, even ergonomics: whereas Monnal T75^®^ (Air Liquide Healthcare) was classified as “ICU-like emergency and transport-ventilators”, Monnal T60^®^ (Air Liquide Healthcare) and Elisée 350^®^ (ResMed) were classified as “sophistical emergency and transport ventilators” [17, 18]. In assist-control mode, three of the four tested transport ventilators both accurately controlled the volume delivered and had acceptable trigger delays in a recent bench study (Michigan test lung); two of the three were the Monnal T60^®^ and Elisée 350^®^ used in our transient ICUs [51]. Despite these bench tests results, using transport ventilators for ARDS management had never been achieved so far in clinical management for such a large number of patients who required a long invasive MV time. As mechanical models can never mimic all complexities of patient-ventilator interactions, short-time bench tests cannot reflect patient evolving lung ARDS mechanistic across time, and they did not allow making direct conclusion on pulmonary consequences (ventilatory lung injuries). Moreover, one must distinguish between ventilator intrinsic performances and capabilities to lung monitoring to avoid shortcut conclusion. However, our results did not show significant in-between groups differences on MV duration or MV outcomes defined as the need for prone positioning, rescue inhaled NO, ECMO for refractory ARDS nor on ventilatory events (ventilator-associated pneumonia). Our data on MV outcomes/events are consistent with previous publication [45].

We do recognize study limitations. First, patients with the greatest severity of critical illness were given priority for admission to the conventional ICU during the first 3 weeks of the epidemic surge. This might be a high risk of selection bias and could influence our study results. However, since end of March 2020 and because of bed availability, the intermediate-bed unit and the post-anesthesia care units operated with direct admissions regardless patients COVID-19 severity, except for those who need renal replacement therapy. Our results did not show significant in-between group difference in terms of ventilator management or severity scores that could have reflect this patient selection bias at ICU admission. The extent to which the more severe critical illness may have cancelled each other out cannot be determined with certainty even if the propensity score matching analysis produced comparable results. Second, the transient ICUs had fewer cubic meters per patient with no wall separations between beds and care was not provided by usual trained ICU teams in the post-anesthesia care units. Thus, being treated with a transport ventilator was associated with many other management differences and it is difficult to conclude that our results are solely a reflection of ventilator type, whereas we did not show worse outcomes in transient ICUs. Third, according to international guidelines and as part of quality process, our physicians are sensitised to lung protective ventilation in case of ARDS management. However, we are not able to provide data for the entire MV duration, which could have highlighted differences between groups. Fourth, our results are based on the evaluation of two types of turbine based non-ICU ventilators and they cannot be generalized to all non-ICU transport ventilators. Fifth, the sample size of 189 patients provided limited statistical power to detect significant outcome differences between the two groups (post-hoc power evaluation, Additional file 1). Sixth, our single center study may not reflect all ICUs in countries that have similar health resources. Finally, we did not assess the association of the ICU overload and mortality based on data on ICU bed capacity and transient ICU expansion.

## Conclusion

In our population of critically ill patients with COVID-19-related ARDS managed at the beginning of the pandemic, ventilator type as one of several differences in ICU care and so management in transient ICU was not associated with mortality, MV duration, or length of stay. Although our study design is not powered to demonstrate any difference in outcome, our results after adjustment do not suggest any signal of harm when using these transport type ventilators as an alternative to ICU ventilators during the three waves of COVID-19 surge. Multicenter studies of larger populations managed based on the current knowledge of COVID-19-related ARDS are needed to further compare associations between ventilator type and patient outcomes.

## Supplementary Information

**Additional file 1: Table S1.** Univariate analysis of factors associated with hospital mortality. **Table S2.** Hospital mortality in ARDS related COVID-19 patients according to ventilator type and matched on a propensity score.

## Data Availability

The data sets used and/or analyzed during the current study are available from the corresponding author on reasonable request.

## Abbreviations

ARDS: Acute respiratory distress syndrome
BMI: Body mass index
COVID-19: Coronavirus disease-2019
CRP: C-reactive protein
ECMO: Extracorporeal membrane oxygenation
ICU: Intensive care unit
IQR: Interquartile range
LDH: Lactate dehydrogenase
MV: Mechanical ventilation
NT-proBNP: NT-pro B-type natriuretic peptide
PCT: Procalcitonin
RT-PCR: Reverse transcriptase-polymerase chain reaction
SAPS II: Simplified Acute Physiology Score II
SARS-CoV2: Severe acute respiratory syndrome-coronavirus 2

## Footnotes

1
http://www.R-project.org. Accessed April 02, 2021.
