# Supplementary material for: Association of ventilator type with hospital mortality in critically ill patients with SARS-CoV2 infection: a prospective study
Source: Ann Intensive Care. 2022 Feb 8;12:10. doi: 10.1186/s13613-022-00981-2 (PMC8821831; doi:10.1186/s13613-022-00981-2)
Supplement: Supplementary file 1 — Additional file 1: Table S1. Univariate analysis of factors associated with hospital mortality. Table S2. Hospital mortality in ARDS related COVID-19 patients according to ventilator type and matched on a propensity score. [file 13613_2022_981_MOESM1_ESM.docx]

**Additional file 1**

**Post-hoc power evaluation**

Given 143 patients exposed to ICU ventilator and 46 patients exposed to transport ventilator, a common survival rate of 32% in both groups (as observed in all patients, n=189), a non-inferiority margin of 15% and a type I error rate of 5%, the statistical power of a non-inferiority study assessing in-hospital survival associated to transport ventilator vs. ICU ventilator, is 29%. Given the in-hospital survival rate as observed in patients matched on propensity score (73% and 70% in patients exposed to transport ventilator and ICU ventilator respectively), the power of the study is 38%. Of note, these results provide the probability we had to keep H0 (the difference in survival rate in patients exposed to ICU ventilator vs. transport ventilator is not higher than 15 %) given our observations, they do not invalidate observations.

**Table S1. Univariate analysis of factors associated with hospital mortality**

|  | | | **Vital status at hospital discharge** | | **Univariate analysis** | | |
| --- | --- | --- | --- | --- | --- | --- | --- |
| **Variables** | | | **Survivors**  N=128 (67.7) | **Non-survivors**  N=61 (32.4) | **OR** | **95%CI** | ***P* value** |
| **Ventilator type** | | |  |  |  |  |  |
| ICU ventilator | | | 95 (74.2) | 48 (78.7) | Ref |  |  |
| Transport ventilator | | | 33 (25.8) | 13 (21.3) | 0.78 | (0.37-1.59) | 0.50 |
| **Demographic characteristics** | | |  |  |  |  |  |
| Age (per years) | | | 63 [55-71] | 71 [62-77] | 1.07 | (1.03-1.10) | <0.001 |
| Male sex | | | 98 (76.6) | 43 (68.9) | 0.68 | (0.34-1.35) | 0.26 |
| Active smokers | | | 8 (6.4) | 3 (5.1) | 0.79 | (0.17-2.85) | 0.74 |
| Coronary artery disease | | | 8 (6.3) | 10 (16.4) | 2.94 | (1.10-8.12) | 0.03 |
| Treated arterial hypertension | | | 58 (45.3) | 29 (47.5) | 1.09 | (0.59-2.02) | 0.77 |
| Diabetes mellitus | | | 34 (26.6) | 21 (34.4) | 1.45 | (0.75-2.79) | 0.27 |
| Immunodeficiency |  | | 8 (6.3) | 10 (16.4) | 2.94 | (1.10-8.12) | 0.03 |
| Obesity (BMI ≥ 30 kg/m^2^) |  | | 52 (40.6) | 18 (30.0) | 0.63 | (0.32-1.19) | 0.16 |
| Respiratory disease (COPD/Asthma/Bronchiectasis) | |  | 19 (14.8) | 8 (13.1) | 0.87 | (0.34-2.05) | 0.75 |
| **Epidemic COVID-19 wave** | |  |  |  |  |  |  |
| Wave 1 | |  | 59 (46.1) | 23 (37.7) | Ref | - | - |
| Wave 2 | |  | 24 (18.8) | 13 (21.3) | 1.39 | (0.60-3.17) | 0.44 |
| Wave 3 | |  | 45 (35.2) | 25 (41.0) | 1.43 | (0.72-2.85) | 0.31 |
| **Characteristics at ICU admission** | |  |  |  |  |  |  |
| Simplified Acute Physiology Score II |  | | 39 [34-45] | 46 [36-56] | 1.06 | (1.03-1.09) | <0.001 |
| Time from symptom onsets to ICU admission (days) | | | 9 [6-12] | 8 [5-10] | 1.01 | (0.95-1.07) | 0.73 |
| Time from hospitalization to ICU admission (days) |  | | 1 [0-3] | 1 [0-4] | 1.08 | (0.98-1.19) | 0.12 |
| Pulmonary co-infection at ICU admission | | | 14 (10.9) | 2 (3.3) | 0.28 | (0.04-1.03) | 0.10 |
| Oxygen requirements at ICU admission (L/min) | | | 15 [15-15] | 15 [15-15] | 0.99 | (0.88-1.12) | 0.85 |
| **Laboratory tests at ICU admission** | | |  |  |  |  |  |
| Lactate (mmol/L) | | | 1.5 [1.2-1.8] | 1.6 [1.3-2.1] | 1.41 | (1.09-1.98) | 0.02 |
| LDH (IU/L) | | | 713 [528-897] | 725 [546-1119] | 1.00 | (1.00-1.00) | 0.05 |
| Lymphocytes (G/L) |  | | 0.68 [0.46-0.96] | 0.62 [0.41-0.91] | 0.98 | (0.82-1.06) | 0.73 |
| C-reactive protein (mg/L) |  | | 163 [97-239] | 183 [90-245] | 1.00 | (1.00-1.00) | 0.44 |
| Procalcitonin (ng/mL) |  | | 0.3 [0.2-0.8] | 0.5 [0.3-2.5] | 1.02 | (0.99-1.05) | 0.22 |
| D-dimers (ng/mL) | | | 1410 [850-2330] | 1750 [1090-3420] | 1.00 | (1.00-1.00) | 0.33 |
| Creatinine (µmol/L) | | | 70 [59-88] | 89 [65-126] | 1.01 | (1.00-1.02) | 0.003 |
| Troponin (ng/mL) | | | 0.01 [0.01-0.02] | 0.02 [0.01-0.06] | 5.57 | (0.62-225) | 0.19 |
| NT-proBNP (pg/mL) | | | 260 [126-552] | 926 [455-2556] | 1.00 | (1.00-1.00) | 0.003 |
| **ICU ventilatory management** | | |  |  |  |  |  |
| High-flow nasal oxygen | | | 74 (57.8) | 38 (62.3) | 1.21 | (0.65-2.27) | 0.56 |
| Non-invasive ventilation | | | 32 (25.0) | 16 (26.2) | 1.07 | (0.52-2.12) | 0.86 |
| Last PaO_2_/FiO_2_ ratio before intubation | | | 90 [71-123] | 86 [72-111] | 1.00 | (0.99-1.01) | 0.82 |
| First PaO_2_/FiO_2_ ratio after intubation | | | 142 [98-182] | 136 [99-165] | 1.00 | (0.99-1.00) | 0.29 |
| Time from ICU admission to oro-tracheal intubation (days) | | | 0 [0-1] | 0 [0-1] | 1.08 | (0.99-1.22) | 0.13 |
| Prone positioning | | | 82 (64.1) | 51 (83.6) | 2.86 | (1.37-6.46) | 0.007 |
| Number of prone position sessions | | | 4 [1-8] | 4 [2-7] | 1.01 | (0.92-1.10) | 0.91 |
| Inhaled nitric oxide | | | 21 (16.4) | 31 (50.8) | 5.27 | (2.68-10.60) | <0.001 |
| ECMO | | | 7 (5.5) | 3 (4.9) | 0.89 | (0.19-3.34) | 0.87 |
| Ventilator associated pneumonia | | | 75 (58.6) | 35 (57.4) | 0.95 | (0.51-1.77) | 0.87 |
| **Adjunctive therapy** | | |  |  |  |  |  |
| Need for vasoactive drugs in the ICU | | | 117 (91.4) | 59 (96.7) | 2.77 | (0.71-18.30) | 0.19 |
| Need for renal replacement therapy in the ICU | | | 12 (9.4) | 16 (26.2) | 3.44 | (1.52-7.99) | 0.003 |
| Dexamethasone initiated at ICU admission | | | 69 (53.9) | 38 (62.3) | 1.41 | (0.76-2.66) | 0.28 |
| **Outcomes** | | |  |  |  |  |  |
| Duration of invasive mechanical ventilation (days) | | | 25 [12-38] | 15 [10-23] | 0.96 | (0.94-0.98) | 0.001 |
| ICU length of stay (days) | | | 29 [16-46] | 17 [12-27] | 0.95 | (0.93-0.97) | <0.001 |
| ICU mortality | | | 0 (0.0) | 60 (98.4) | - |  |  |
| Hospital mortality | | | 0 (0.0) | 61 (100.0) | - |  |  |

Data are presented as N (%) or Median [interquartile range]

OR = Odd Ratio; 95% CI = 95% confidence interval; BMI = body mass index; COPD = chronic obstructive pulmonary disease; ICU = intensive care unit; LDH = lactate deshydrogenase; NT-proBNP = NT-pro B-type natriuretic peptid; ECMO = extracorporeal membrane oxygenation.

**Table S2: Hospital mortality in ARDS related COVID-19 patients according to ventilator type and matched on a propensity score^a^**

|  | **N (%) or Median [Interquartile Range]** | |  |
| --- | --- | --- | --- |
|  | **ICU ventilator**  **n = 70 (63.0)** | **Transport ventilator**  **n =41 (37.0)** | ***P* value** |
| **Hospital mortality** | **21 (30.0)** | **11 (26.8)** | **0.83** |
| Age | 66 [58-74] | 64 [60-70] | 0.53 |
| Male sex | 50 (71.4) | 26 (63.4) | 0.40 |
| Time from hospitalization to ICU admission | 1 [0-3] | 1 [0-3] | 0.56 |
| SAPS II | 40 [35-46] | 40 [35-45] | 0.68 |
| Respiratory SOFA score | 3 [3-4] | 3 [3-4] | 0.70 |
| Hepatic SOFA score | 0 [0-0] | 0 [0-0] | 0.64 |
| Creatinine at ICU admission  ≥ 100 µmol/L  < 100 µmol/L | 9 (12.9)  61 (87.1) | 7 (17.1)  34 (82.9) | 0.58 |
| LDH value at ICU admission | 661 [481-855] | 635 [487-795] | 0.42 |
|  |  |  |  |

^a^ The propensity score used age, male, sex, time from hospitalization to ICU admission, Simplified Acute Physiology Score II (SAPS II), respiratory and hepatic SOFA score, creatinine ≥ 100 µmol/L and LDH value at ICU admission.
